# Telemedicine for Gestational Diabetes Mellitus (TeleGDM): A Mixed-Method Study Protocol of Effects of a Web-Based GDM Support System on Health Service Utilization, Maternal and Fetal Outcomes, Costs, and User Experience

**DOI:** 10.2196/resprot.6044

**Published:** 2016-08-09

**Authors:** Tshepo Mokuedi Rasekaba, Kwang Lim, Irene Blackberry, Kathleen Gray, John Furler

**Affiliations:** ^1^ Department of General Practice The University of Melbourne Carlton Australia; ^2^ Department of General Medicine The Northern Hospital Epping Australia; ^3^ Department of Medicine The University of Melbourne Parkville Australia; ^4^ Royal Melbourne Hospital Department of Medicine and Aged Care Parkville Australia; ^5^ John Richards Initiative College of Science, Health and Engineering La Trobe University Wodonga Australia; ^6^ Health and Biomedical Informatics Centre The University of Melbourne Parkville Australia

**Keywords:** gestational diabetes, telemedicine, Internet, electronic personal health record

## Abstract

**Background:**

Women with insulin-treated gestational diabetes mellitus (GDM) require close monitoring and support to manage their diabetes. Recent changes to the diagnostic criteria have implications for service provision stemming from increased prevalence, suggesting an increased burden on health services in the future. Telemedicine may augment usual care and mitigate service burdens without compromising clinical outcomes but evidence in GDM is limited.

**Objective:**

The Telemedicine for Gestational Diabetes Mellitus (TeleGDM) trial aims to explore the use of telemedicine in supporting care and management of women with GDM treated with insulin.

**Methods:**

The TeleGDM is a mixed-methods study comprising an exploratory randomized controlled trial (RCT) and a qualitative evaluation using semistructured interviews. It involves women with insulin-treated GDM who are up to 35 weeks gestation. Participating patients (n=100) are recruited face-to-face in outpatient GDM clinics at an outer metropolitan tertiary hospital with a culturally diverse catchment and a regional tertiary hospital. The second group of participants (n=8) comprises Credentialed Diabetes Educator Registered Nurses involved in routine care of the women with GDM at the participating clinics. The RCT involves use of a Web-based patient-controlled personal health record for GDM data sharing between patients and clinicians compared to usual care. Outcomes include service utilization, maternal and fetal outcomes (eg, glycemic control, 2nd and 3rd trimester fetal size, type of delivery, baby birth weight), diabetes self-efficacy, satisfaction, and costs. Semistructured interviews will be used to examine user experiences and acceptability of telemedicine.

**Results:**

The trial recruitment is currently underway. Results are expected by the end of 2016 and will be reported in a follow-up paper.

**Conclusions:**

Innovative use of technology in supporting usual care delivery in women with GDM may facilitate timely access to GDM monitoring data and mitigate care burdens without compromising maternal and fetal outcomes. The intervention may potentially reduce health service utilization.

**Trial Registration:**

Australian and New Zealand Clinical Trials Registry (ANZCTR): ACTRN12614000934640; https://www.anzctr.org.au/Trial/Registration/TrialReview.aspx?id=366740 (Archived by WebCite® at http://www.webcitation.org/6jRiqzjSv).

## Introduction

Recent changes to tighten the diagnostic criteria for gestational diabetes mellitus (GDM) [1] mean many more women will be diagnosed with this condition, placing increased demand on clinical services to provide diabetes care. Women with insulin-treated GDM, in particular, often require more intensive follow-up and support for titration of insulin and overall management of GDM [2,3].

The prevalence of GDM is estimated to be 6% to 15% of pregnancies [1,4] dependent on whether the diagnostic criteria set by the International Association of Diabetes and Pregnancy Study Groups (IADPSG) or the Australasian Diabetes in Pregnancy Society (ADIPS) is used. The IADPSG’s revisions in recent years give higher prevalence estimates [1].

Good control of blood glucose level (BGL) in GDM is important to minimize the risk of pregnancy and birth complications associated with the condition. Such complications can include large for gestational age (LGA) babies, macrosomia, increased likelihood of cesarean delivery, preeclampsia, and fetal shoulder dystocia [3-5]. First-line therapy to control hyperglycemia involves dietary modification and physical activity [1,3,6] or oral hypoglycemic agents (OHA) [3]. An insulin regimen is initiated if the OHA therapies are inadequate in optimizing BGL or there is evidence of increased risk of macrosomia [6]. Approximately 50% of women with GDM go on insulin regimen, which requires close monitoring and intensive follow-up for regular insulin titrations to control persisting hyperglycemia [2].

The increasing prevalence of GDM [7-10] and the intensive clinical care needed have implications for the capacity of health care services to provide timely care and the clinical outcomes of such care. There is a need to explore innovative ways to deliver care and support for women with GDM to ease the service burden while not compromising quality of care. This may also potentially deliver cost efficiencies and savings.

In our systematic review [11], telemedicine has emerged as a potentially effective intervention to address service utilization while producing maternal and fetal outcomes similar to or better than usual care.

Telemedicine is defined as “the use of telecommunications technology to provide medical information and service” [12]. Telemedicine (also known as telehealth) has been implemented as a monitoring intervention in diabetes, heart failure, and chronic obstructive pulmonary disease [9,10,13] with promising results. For instance, a small study that trialled the use of cellular phones to transmit self-monitoring blood glucose data in type 2 diabetes found the approach was feasible, easy to use, and resulted in patients having fewer hospital visits [8]. Recent studies exploring a smartphone application or text messaging in type 1 and/or 2 diabetes reported improvements in glycemic control in favor of the telehealth approaches [14,15], while self-efficacy and quality of life were unchanged [14]. A study of telemedicine in heart failure patients reported better quality of life and heart failure self-care while hospital utilization remained unchanged [13]. While there may be some cautious optimism about the benefits of telehealth-based interventions, usage by patients appears modest, approximately 34% to 39% [16]. It remains to be seen how all this translates to GDM, especially in a real-world clinical setting.

Specifically in GDM, telemedicine interventions compared to control/usual care may reduce service utilization such as face-to-face clinic visits (4.25 [standard deviation or SD 0.93] vs 6.22 [SD 1.48], respectively; *P*=.002) and unscheduled visits (0.50 [SD 0.73] vs 2.89 [SD 1.05], respectively; *P*<.001) [12], while achieving similar outcomes (with trends in favor of telemedicine) for glycemic control, birth weight, incidence of macrosomia [11,12,17,18] and diabetes self-efficacy [12,17-19]. The main limitations of studies of telemedicine in GDM that we identified in our systematic review of the literature [11] are that there are very few randomized controlled trials (RCT) and sample sizes tend to be small. None of the trials included in our review evaluated costs, perhaps due to the lack of an agreed standardized evaluation framework for telehealth interventions. We also identified other methodological limitations such as shorter interventions and the heterogeneous nature of the outcomes and telehealth interventions used [10,20-22]. Interventions were perhaps too short to have significant measurable impacts; outcome measures varied across studies, posing challenges to conducting effects through pooled data analysis, and the interventions varied considerably, ranging from telephone support and videoconferencing to text messaging [10,20-22], making comparison of studies and generalizability difficult.

Our innovative study, the Telemedicine for Gestational Diabetes Mellitus (TeleGDM) trial, uses a Web-based approach to augment the management of women with insulin-treated GDM. Our aim is to explore the effects of telemedicine on health system performances including patient utilization of outpatient clinical care, maternal and fetal clinical health outcomes, and patient and clinician satisfaction and acceptance with respect to the intervention technology. In addition, a cost comparison between the two arms of the trial will be performed to determine if there are any provider cost savings that might be associated with changes in outpatient clinic attendance.

We hypothesize that with timely access to patient GDM self-monitoring data, health service utilization would be decreased without compromising maternal and fetal outcomes with an associated provider cost saving, greater satisfaction with the telemedicine, and a positive user experience.

The project is registered with the Australian and New Zealand Clinical Trial Registry [ACTRN12614000934640], and ethics approval was granted by Northern Health Human Research Ethics Committee (HREC P/11/14) and Bendigo Health Human Research Ethics Committee (HREC/15/BHCG/44).

## Methods

### Study Design

**Figure 1:**
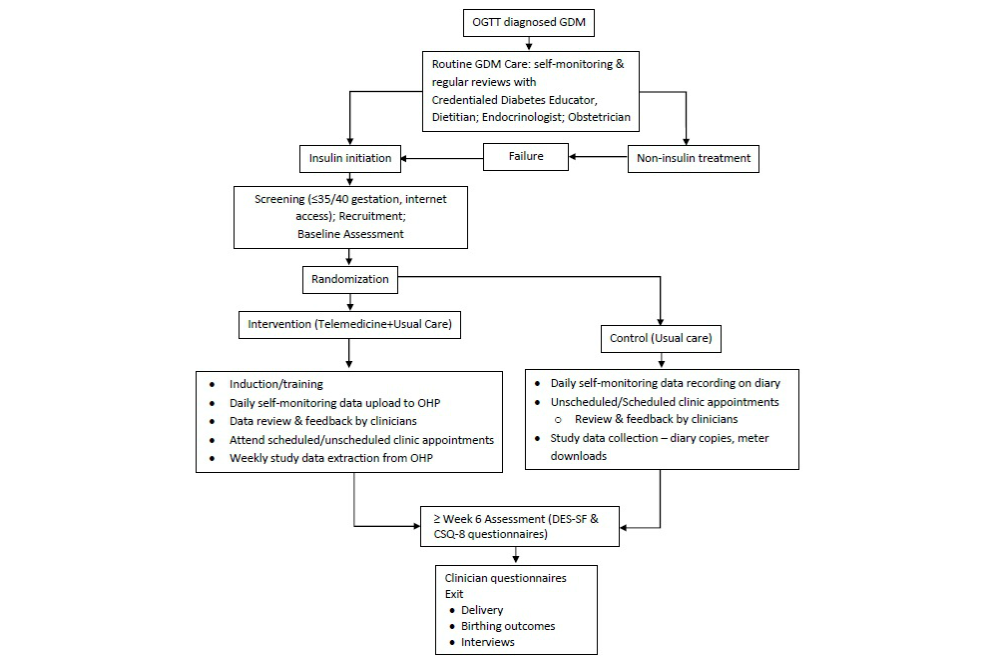
Study design flowchart.

The TeleGDM trial is a mixed-methods study comprising an exploratory RCT and a qualitative evaluation using semistructured interviews (Figure 1). RCTs are the gold standard for providing evidence for practice [23,24] but have a major limitation of “. . . not tell(ing) the whole story . . . ” [25]. Qualitative methods such as interviews can provide more in-depth information about participant experiences [26] than would otherwise be captured by quantitative methods alone.

### Population, Setting, and Inclusion Criteria

The first group of participants comprises pregnant women diagnosed with GDM who have commenced insulin therapy to control hyperglycemia. These women attended outpatient GDM clinics at two tertiary hospitals between August 30, 2014, and October 30, 2016, inclusive of follow-up. One hospital is in an outer metropolitan region with a catchment population of significant cultural and linguistic diversity. The other is regionally located and serves a population with a rural background. Combined, the two hospitals have approximately 5000 live singleton births annually, and approximately 800 of the pregnancies are affected by GDM.

Women with GDM (patient participant group) are eligible for inclusion if they have a clinical diagnosis of GDM based on the IADPSG criteria following an oral glucose tolerance test [1]. Other eligibility criteria include gestation up to 35 weeks and access to the Internet via a personal computer, smartphone, or tablet. Prepregnancy glucose intolerance, twin pregnancies, GDM not treated with insulin, and other types of diabetes are exclusion factors.

The second group of participants are Credentialed Diabetes Educator Registered Nurses (CDE-RNs) who provide GDM care at the two centers. The CDE-RNs are directly involved in the RCT component of the study and provide care to women with GDM in the course of their usual practice. The number of these clinicians across the two sites is 8; all are requested to complete the clinician assessments for the study.

### Recruitment and Randomization

The women with GDM regularly attend outpatient GDM clinics at the hospitals. It is at these weekly clinics that prospective participants are recruited face-to-face. Clinicians identify potentially eligible patients, give them a study brochure and/or seek permission for referral to the lead researcher or study research assistants. Following referral, participants are approached for face-to-face screening, detailed briefing, consent, randomization, and completion of baseline questionnaires. A 1:1 randomization schedule was generated in STATA 11.0 (StataCorp LP) by an independent statistician. The lead researcher and RAs have no involvement in routine care of the patients.

Some ethnic groups (Indian, Asian, Arabic/Middle Eastern, Pacific Islander, Aboriginal, and African) are considered high risk for GDM [27]. Previous GDM and use of insulin in past pregnancies are also considered high risk factors for GDM. Therefore randomization was stratified according the level of risk (high or low). Stratification avoids group allocation imbalances on factors that have significant influence on prognosis, avoids type 1 error, and improves study power for small trials [28]. Group assignments are concealed in two sets of opaque envelopes; the first set is the randomization schedule for the low risk subgroup and the second for the high risk GDM subgroup. Following consent, the envelopes are consecutively opened for assignment by the recruiter. Clinicians are not blinded to group allocations because they need data from the intervention for clinical care.

### Usual Care (Control)

Usual care refers to clinical GDM care processes currently in practice at the participating hospitals, and this will be the control group. In line with recommended best practice [29,30] diagnostic screening for GDM occurs at 24 to 28 weeks gestation for women with no known history of diabetes or earlier for those considered high risk for GDM. Following diagnosis through to end of pregnancy, ongoing care is provided via a multidisciplinary team of endocrinologists, dietitians, and CDE-RNs. The role of the team is in addition to obstetric care.

From an endocrinology perspective, care involves an initial group counseling and education with a CDE-RN and dietitian covering aspects of GDM self-management. The CDE-RNs provide the pregnant women with free BGL meters from an approved supplier. The meters are individual use and the women purchase their own consumables (ie, test strips and lancing devices). Treatment targets are ≤5.0 mmol/L for preprandial BGL and ≤6.7 mmol/L for 2-hour postprandial BGLs. Insulin is initiated or titrated if BGLs are above target over three successive days. Ongoing face-to-face appointments are scheduled with members of the team as needed until delivery. Appointments generally occur every one to two weeks as determined by the clinicians. Patients on insulin have more frequent reviews especially in the early stages of insulin initiation. Self-management involves keeping a daily paper diary record of GDM self-monitoring data (1 preprandial and 3 postprandial BGLs, insulin dosing, symptoms, and dietary information). The diaries are reviewed by the clinicians at each outpatient clinic. The women also have the option to call the CDE-RNs out of scheduled appointments if BGLs are outside target.

### Telemedicine (Intervention)

The intervention is telemedicine as an adjunct to usual care. The main distinction to usual care is GDM self-monitoring data is shared via a telemedicine system in lieu of paper diaries. The intervention uses a Web-based portal, Online Health Portfolio (OHP) [31], for data sharing and communication between patients and clinicians and is premised upon (1) women with GDM undertaking regular GDM self-monitoring and entering data; (2) timely availability of data to clinicians via the Web-based OHP, hence timely response to the women’s GDM care needs informed by the available data; and (3) upon carrying out advice and feedback the women will better manage GDM and require less frequent appointments. Currently there is no empirical evidence for OHP, and it was chosen for this study on pragmatic reasons and anecdotal accounts of independent endocrinologists who used it in their practice.

Online Health Portfolio is a secure Web-based patient-controlled personal health record that is accessed securely through an Internet browser on a personal computer, smartphone or tablet. It is a proprietary system developed and owned by a vendor who is independent of the study. It uses 256-bit data encryption and 5-minutes inactivity time logout. Besides data entry and preview, the users can have graphical visualization of summary data and trends filtered by pre- or postprandial meal type or time, set up automatic reminders on the internal calendar, and set trigger levels for BGL alerts. Reminders may be forwarded to the patient’s smartphone as a short message service (SMS) text. There is an internal messaging feature within OHP to enable 2-way messaging of free text between clinicians and patients. Clinicians also have the option to send an SMS text to the patient’s smartphone from OHP. Participating patients use their own Internet-connected devices while clinicians use their usual hospital-provided Internet-connected computers. Username and password access to OHP is independent of all other hospital applications and systems. While patients are at liberty to access OHP at any time, clinicians interact with OHP during the course of normal work hours (8:00 AM to 4:30 PM), Monday through Friday. Multimedia Appendix 1 and Multimedia Appendix 2 show of some screenshots of the OHP.

**Figure 2:**
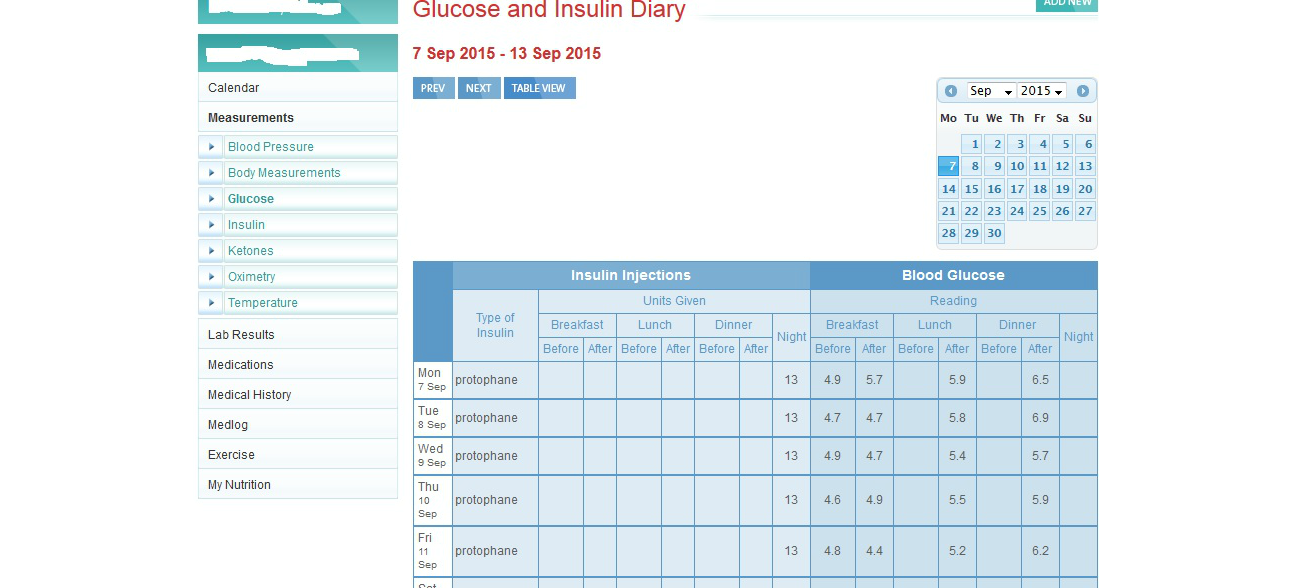
Diary view format introduced after modifications to enhance usability of OHP.

The research team have no financial interest in the OHP. The lead researcher (TR) has had some input into modifications and refinements to the Web portal in order to enhance usability by the patients and clinicians. An example is the introduction of the diary view format in Figure 2. The vendor usually charges an annual subscription fee (AUD $85) to patients to use OHP while clinicians’ subscriptions are free. For this study, patient subscriptions are covered in the study budget. OHP consumes negligible amounts Internet data, thus adding no perceptible costs to patients’ own home or mobile Internet service.

Upon enrollment, patient participants undergo individual semistructured 30 to 45 minutes induction by the lead researcher or research assistants.

The induction is hands-on and covers the initial set-up with participants practicing all the tasks they are expected to perform independently from then on. Induction covers signing up, logging on, navigating through the OHP Web portal, data entry, messaging, and reviewing data trend/summary graphs. All data entry is practiced using the previous day’s data. Performing BGL self-monitoring, administering insulin, and following dietary advice are part of routine diabetes education and counseling provided by a multidisciplinary endocrinology care team as described under usual care. Participants are also instructed on how to share this health information with the GDM clinicians for the purpose of providing clinical care and with the project lead investigator for research data collection and data management purposes. When required and in order to improve study data collection, the lead researcher may set up automatic reminders on OHP to send reminders every second day to prompt the noncomplying patient to enter data. Activating or setting up automated reminders is not routine but it is targeted for those who fail to perform data entry according to expectations. This avoids inundating those who are compliant with unnecessary reminders.

Participants are asked to enter their GDM self-monitoring data onto OHP daily or every other day in order to minimize backlogs and associated data entry errors. Maintaining a paper diary is optional. Automated alerts about new data entries are sent to the clinicians via email prompting the clinicians to log in under their credentials to review the patient data. When required and depending on the reviewed data or patient queries, clinicians provide feedback to the patient via the messaging service about any necessary alterations to treatment (eg, insulin titrations, changes to diet). The CDE-RNs act as the gatekeepers to interact with the telemedicine system and to consult or liaise with other GDM service team members. Patients can also email or print reports for other interested parties who do not have direct access to the Web-based shared data.

Induction for clinicians involved in providing care was conducted by the lead researcher. It consisted of setting up log-on credentials, using and navigating through the OHP webpage, setting up alerts, reviewing patient data, and messaging. The induction included both demonstration and hands-on practice in group setting.

Tasks expected of clinicians are to review patient data at their convenience, fitting in with their other routine clinical commitments through the day during weekdays. At the minimum, data are reviewed every 1 to 2 days during the week. Clinical decision making and advice in relation to ongoing management of GDM is at the discretion of the clinicians in accordance with existing clinical protocols without interference from the researchers. The same applies to scheduling of clinic appointments. Clinicians may also remind a patient when no data have been entered.

For research data collection, participant engagement, and/or troubleshooting purposes, the lead researcher periodically contacts participants via the OHP messaging feature or telephone and extracts all data from OHP to collate in a secure MS Access study database. The lead researcher is the primary contact for basic technical support queries, escalating any queries that cannot be resolved to the OHP vendor.

### Sample Size

As an exploratory RCT, a stringent sample size calculation was deemed to be less critical for the TeleGDM trial. Therefore sample size has been set at 100 participants. This determination was largely pragmatic, based on resources, time constraints, the balance of probability for detecting a statistically significant difference in the primary outcome and a reasonable power for secondary outcomes. Estimations based on a finding of 44% fewer clinic visits among those receiving telemedicine versus controls [32] indicated a required sample size of 42 with a power of 0.9 for a similar outcome. Thus if the primary outcome in our study were to be less than the latter cited study, or there was 30% attrition, our set target sample offers good prospects for detecting a difference in the primary outcome.

### Data and Outcomes

Data for research is collected by the lead researcher. This includes weekly extraction of data from OHP for those in the intervention arm in addition to questionnaire outlined below. For controls, photocopies of patients’ paper diaries are obtained when these patients attend their clinic appointments. In addition to these photocopies, where possible, BGL data are directly extracted from the BGL meter via USB cable connection. Finally, once patients have reached the study end point, they also asked to send outstanding self-monitoring data copies of their diaries via email or as photos via smartphone-based multimedia messaging service.

Demographic data together with diabetes self-efficacy and client satisfaction are collected at baseline with follow-up at least 6 weeks after enrollment in the trial. Self-efficacy and satisfaction are measured using the Diabetes Empowerment Scale–Short Form (DES-SF) [33] (Multimedia Appendix 3) and Client Satisfaction Questionnaire–8 Item (CSQ-8) [34,35] (Multimedia Appendix 4). The DES-SF is a shorter version of the original 28-item questionnaire for measuring self-efficacy in people with insulin- or noninsulin-treated diabetes [36]. The original questionnaire has three subscales: managing the psychosocial aspects of diabetes, assessing dissatisfaction and readiness to change, and setting and achieving goals. The longer version has high construct validity and good reliability [18,36]. The shorter version has 8 items, has high reliability (alpha of 0.85), and the scores were found to change positively with improvement in HbA_1c_ [33]. To minimize the burden on participating women we selected the DES-SF to assess diabetes self-efficacy. The CSQ-8 has been used in diabetes research [37] and was assessed for reliability and validity in a childbirth service evaluation [38]. It is reported to have strong reliability, excellent face validity [34,35], good psychometric properties, high client and staff acceptability, and sensitivity to programs of varying quality [37]. The CSQ-8 is available under paid license while the DES-SF is free with appropriate attribution. Both the DES-SF and SCQ-8 questionnaires are self-completed face-to-face or administered over the phone at baseline and at least six weeks from enrollment. Further information on outcomes and data collection time points is provided in Table 1.

The primary outcome of the quantitative exploratory RCT component of the study is service utilization. Maternal and fetal outcomes, satisfaction, and costs are secondary outcomes. In particular, one of the limitations of studies in our systematic review [11] was the lack of cost evaluation, however basic. Considering that studies appear to show virtually similar clinical outcomes between telemedicine and usual care/control [12,17-19], a form of cost comparison becomes important. There are several methods for undertaking health economic evaluation, one of which is cost minimization. This type of health economic evaluation is defined as “. . . evaluation method to use when the case for an intervention has been established and the programmes or procedures under consideration are expected to have the same, or similar, outcomes. In these circumstances, attention may focus on the cost side of the equation to identify the least costly option” [39]. At the time of this protocol study, patients not covered by the Australian Medicare paid AUD $280 for each face-to-face consultation with a clinician for GDM care at the centers in this study. Because diabetes education, endocrinology, and dietetics are the key outpatient specialties involved directly in GDM management and therefore targeted for influence by the TeleGDM intervention, the AUD $280 cost rate will be assigned to these for service provided to patients between study entry and study exit. Study participant outpatient consultations data covering service access between commencement of recruitment and end of data collection for computation of provider costs will be sourced from the hospital data management unit. While clinicians provide service over the phone, which is a cost to the hospital, patients are not billed and hence this cost will not be included. Furthermore, implementing the intervention required existing equipment and infrastructure for the brief induction. Costs for these were considered negligible and therefore were not taken into consideration. Also Australian public hospitals by their nature are nonprofitmaking entities. Where fees are charged these are normally break-even and include overheads. Besides the AUD $85 per patient subscription there is no separate license fee for OHP.

Technology is central to the telemedicine support service for GDM and an important feature for evaluation. As such, technology capability will be assessed through the volume of data uploads by patients and qualitatively through sections 2 and 3 (system and information quality) of the Health Infoway System and Use Assessment Survey [40] (Multimedia Appendix 5).

Outcomes of interest are outlined in Table 1. These are aligned with the dimensions of the telehealth evaluation framework proposed by the Institute for a Broadband-Enabled Society [41-43].

**Table 1:** Outcomes and indicators matched to the telehealth evaluation framework dimensions.

	Outcome	Telehealth evaluation framework dimension	Measures/ indicators	Assessment instrument/ data source	Time point
**Primary**					
	Patient service utilization	Patient control	Number of scheduled face-to-face consultations	Attendances and nonattendances from outpatient activity dataset; patient medical records	Study exit
			Number of unscheduled face-to-face consultations		
			Number of telephone consultations		
**Secondary**					
	Clinical measures and satisfaction	Clinician quality of care	Glycemic control	BGL^a^ extraction from OHP^b^; glucometer downloads; patient paper diaries	Enrollment; delivery
			Glycemic stability	Time (days) to BGL stabilization	Between enrollment and delivery
			Insulin adjustments	Time (days) between insulin adjustment	Between enrollment and delivery
			Macrosomia	Fetal ultrasound biometry	2nd (17-22 weeks) and 3rd (>22 wks) trimester
			Diabetes self-efficacy	DES-SF^c^	Baseline; ≥6 weeks
			LGA^d^	Birth weight > 90th percentile	Delivery
			Neonate admission to SCN^e^	Patient medical record	Delivery
			Type of delivery (NVD^f^, LUSCS^g^, other)	Patient medical records	Delivery
			Mother/patient satisfaction with clinical care	CSQ-8^h^	Baseline; ≥6 weeks
					Enrollment; delivery
	Costs	Organization sustainability	Service provider costs	Routine billing administrative data for face-to-face/staff costs; OHP subscriptions	Study exit
**Tertiary**					
	Usage (patients and clinicians)	Technology capability	Clinician system, information and service quality, usage	Modified Canada Health Infoway System And Use Assessment Survey	6 months from beginning of study
			OHP access; volume of data uploaded	Extraction from OHP logs	Study completion

^a^BGL: blood glucose level

^b^OHP: Online Health Portfolio

^c^DES-SF: Diabetes Empowerment Scale–Short Form

^d^LGA: large for gestational age

^e^SCN: special care nursery

^f^NVD: normal vaginal delivery

^g^LUSC: lower uterine segment cesarean section

^h^CSQ-8: Client Satisfaction Questionnaire–8 Item (CSQ-8)

### Qualitative Evaluation

The aim of the qualitative evaluation is to supplement the RCT by exploring patient and clinician acceptance, adoption, and experiences of telemedicine to support care in the management of GDM. A semistructured interview approach is used for both patient and clinician participants. The interview schedule is outlined in Multmedia Appendix 6. Subjects include those who are assigned to the intervention arm of the TeleGDM RCT and the CDE-RNs. A purposive sample of patients will be selected with the aim for up to 15 patients. Since there are only a few clinicians, all CDE-RNs who actively interact with OHP during the RCT will be included. Interviews are conducted by the lead researcher and the questions are open-ended, focusing on gathering interviewee experiences with telehealth-supported GDM management and the technology under use. Clinician interviews are face-to-face while patient interviews are carried out over the phone for the convenience of new mothers. All interviews are audiorecorded digitally for later verbatim transcription.

The interviews will be supplemented with field notes/observations. Notes or written diaries throughout the trial allow for an analysis that provides a narrative account of practice [44]. The narrative adds to the evaluation by highlighting factors in the local setting which may influence the success or failure of the intervention [44,45].

### Data Preparation and Analysis

Quantitative data analysis will be performed using Stata/IC 13.1 (StataCorp LP) with an intention to treat analysis. Missing data for the primary outcome is expected to be minimal as all patient appointments and outcomes are recorded. For the DES and CSQ-8 losses to follow-up will employ last observation carried forward for missing values. Since case BGL data is serial and expected to be nonlinear, case mean of nearby data points imputation will be used for missing data.

Summary univariate statistics will be used to describe the study populations and compare study groups at baseline. Categorical variables will be summarized as raw numbers and percentages and between groups comparisons will utilize chi-square statistics. Multivariate statistical analysis will be performed to compare the groups on primary and secondary outcomes. In addition, survival analysis will be performed to explore time to reach glycemic stability. Statistics will be reported with standard deviations or 95% confidence intervals as appropriate. Statistical significance will be indicated by *P*<.05.

Patient and clinician interviews will undergo thematic analysis supported by NVivo 11 (QSR International). The interview transcripts will be analyzed separately for each participant group using an inductive approach to identify and/or infer themes and codes from the transcripts. Further themes will be classified according to the dimensions of telehealth evaluation framework [41].

## Results

At the time of submission of this paper, recruitment and data collection were underway. Data analysis was pending and results expected at the end of 2016.

## Discussion

### Summary

Use of telemedicine to support care, specifically in the management of GDM, through a multiplatform Web-based personal health record is an innovative use of current technologies. It is envisaged the study will show reductions in health care utilization (eg, face-to-face clinic appointments) with an associated service provider cost saving. Other expected effects are GDM clinical outcomes similar to if not better than usual care. In addition, it is anticipated that both clinicians and patients will express greater satisfaction, usability, and positive views for telemedicine-supported GDM management.

The increasing prevalence of GDM and associated burdens [1-3] calls for innovative ways of service provision. The TeleGDM study explores a Web-based telemedicine approach to providing care and support to pregnant women with insulin-treated GDM. The intervention in the TeleGDM study relies on reliable and acceptable technology for efficient data sharing between patients and clinicians. Underpinning the intervention is the idea that telemedicine provides an engagement and interaction platform between the patient and clinician independent of face-to-face visits. The intervention incorporates some of the elements which are common for Web-based interventions (eg, self-management, communication, individualized feedback) [46].

### Comparison With Previous Work

A few previous studies [12,17-19] have specifically explored telemedicine for GDM. These studies found better service utilization in terms of fewer face-to-face appointments and better diabetes psychological self-efficacy. There are some marked differences between the TeleGDM study and previous studies; TeleGDM uses technologies (broadband Internet and the ubiquitous mobile telephony Internet) which were not previously available. While in theory the approach in the TeleGDM study appears similar to those in the previous studies, the intervention has been implemented as adjunct to usual care for ethical reasons. That is, usual care is the current standard of care at the study sites, and therefore it would be unethical to deny patients what is current practice in lieu of a test intervention. However, the adjunct nature of the intervention means concurrent elements of usual care could become confounders.

### Strengths and Limitations

The strength of the TeleGDM is the innovative use of current technologies in GDM, particularly in the Australian context. Second, the study uses a mixed-method approach to enhance the rigor of the evaluation and incorporates elements of a framework proposed for evaluating telehealth interventions in Australia [41]. The study includes cost evaluation, an important consideration which telehealth studies are often criticized for excluding [10]. Costs are only considered from a provider perspective and limited to billable consultations for pragmatic reasons, a potential methodological limitation. As such, a full economic evaluation that takes into account other costs could be a future consideration.

Internet security is one of the barriers to uptake of Web-based interventions [46]. Hence OHP uses 256-bit data encryption, individual username and password access, and an inactivity timeout. Despite these security measures, data breaches cannot be completely ruled out. Any interactions over the Web carry the risk that user privacy and confidentiality may be breached, however minimal. This may happen as a result of unauthorized access during the course of transmission, hacking into system servers, or users not exercising due diligence in securing their log-on information.

### Conclusion

TeleGDM is an innovative use of technology to support care and management of insulin-treated GDM. It may mitigate burdens on the health care service and the women with GDM without compromising clinical outcomes. Results of this study are expected by the end of 2016.

## Appendix

Multimedia Appendix 1 Online Health Portfolio data entry screenshots.

Multimedia Appendix 2 Online Health Portfolio data view screenshots.

Multimedia Appendix 3 Diabetes Empowerment Scale--Short Form (DES-SF).

Multimedia Appendix 4 Client Satisfaction Questionnaire-8 Item (CSQ-8).

Multimedia Appendix 5 Canada Health Infoway System and Use Assessment Survey.

Multimedia Appendix 6 Clinician and patient interview schedule.

## Abbreviations

ADIPS: Australasian Diabetes in Pregnancy Society
CDE-RN: Credentialed Diabetes Education–Registered Nurse
CSQ-8: Client Satisfaction Questionnaire–8 Item (CSQ-8)
DES-SF: Diabetes Empowerment Scale–Short Form
GDM: gestational diabetes mellitus
IADPSG: International Association of Diabetes and Pregnancy Study Groups
LGA: large for gestational age
OHP: Online Health Portfolio
RCT: randomized controlled trial

## References

[1] Moses RG, Morris GJ, Petocz P, San GF, Garg D (2011). The impact of potential new diagnostic criteria on the prevalence of gestational diabetes mellitus in Australia. Med J Aust.

[2] Wong VW, Jalaludin B (2011). Gestational diabetes mellitus: who requires insulin therapy?. Aust N Z J Obstet Gynaecol.

[3] Landon MB, Gabbe SG (2011). Gestational diabetes mellitus. Obstet Gynecol.

[4] Cheung NW, Byth K (2003). Population health significance of gestational diabetes. Diabetes Care.

[5] Crowther CA, Hiller JE, Moss JR, McPhee AJ, Jeffries WS, Robinson JS, Australian Carbohydrate Intolerance Study in Pregnant Women (ACHOIS) Trial Group (2005). Effect of treatment of gestational diabetes mellitus on pregnancy outcomes. N Engl J Med.

[6] Hoffman L, Nolan C, Wilson JD, Oats JJ, Simmons D, Australasian Diabetes in Pregnancy Society (1998). Gestational diabetes mellitus--management guidelines. Med J Aust.

[7] Weinstock RS, Teresi JA, Goland R, Izquierdo R, Palmas W, Eimicke JP, Ebner S, Shea S (2011). Glycemic control and health disparities in older ethnically diverse underserved adults with diabetes: five-year results from the Informatics for Diabetes Education and Telemedicine (IDEATel) study. Diabetes Care.

[8] Geisler E, Wickramasinghe N (2009). The role and use of wireless technology in the management and monitoring of chronic diseases.

[9] Fursse J, Clarke M, Jones R, Khemka S, Findlay G (2008). Early experience in using telemonitoring for the management of chronic disease in primary care. J Telemed Telecare.

[10] Wootton R (2012). Twenty years of telemedicine in chronic disease management--an evidence synthesis. J Telemed Telecare.

[11] Rasekaba TM, Furler J, Blackberry I, Tacey M, Gray K, Lim K (2015). Telemedicine interventions for gestational diabetes mellitus: A systematic review and meta-analysis. Diabetes Res Clin Pract.

[12] Pérez-Ferre N, Galindo M, Fernández MD, Velasco V, Runkle I, de la Cruz MJ, Martín RP, Del VL, Calle-Pascual AL (2010). The outcomes of gestational diabetes mellitus after a telecare approach are not inferior to traditional outpatient clinic visits. Int J Endocrinol.

[13] Seto E, Leonard K, Cafazzo J, Barnsley J, Masino C, Ross H (2012). Mobile phone-based telemonitoring for heart failure management: A randomized controlled trial. J Med Internet Res.

[14] Kirwan M, Vandelanotte C, Fenning A, Duncan MJ (2013). Diabetes self-management smartphone application for adults with type 1 diabetes: randomized controlled trial. J Med Internet Res.

[15] Dobson R, Carter K, Cutfield R, Hulme A, Hulme R, McNamara C, Maddison R, Murphy R, Shepherd M, Strydom J, Whittaker R (2015). Diabetes Text-Message Self-Management Support Program (SMS4BG): A Pilot Study. JMIR mHealth uHealth.

[16] Holmen H, Torbjørnsen A, Wahl AK, Jenum AK, Småstuen MC, Arsand E, Ribu L (2014). A mobile health intervention for self-management and lifestyle change for persons With type 2 diabetes, part 2: one-year results from the Norwegian randomized controlled trial RENEWING HEALTH. JMIR Mhealth Uhealth.

[17] Homko CJ, Deeb LC, Rohrbacher K, Mulla W, Mastrogiannis D, Gaughan J, Santamore WP, Bove AA (2012). Impact of a telemedicine system with automated reminders on outcomes in women with gestational diabetes mellitus. Diabetes Technol Ther.

[18] Homko CJ, Santamore WP, Whiteman V, Bower M, Berger P, Geifman-Holtzman O, Bove AA (2007). Use of an internet-based telemedicine system to manage underserved women with gestational diabetes mellitus. Diabetes Technol Ther.

[19] Pérez-Ferre N, Galindo M, Fernández MD, Velasco V, de la Cruz MJ, Martín P, del Valle L, Calle-Pascual AL (2010). A telemedicine system based on Internet and short message service as a new approach in the follow-up of patients with gestational diabetes. Diabetes Res Clin Pract.

[20] McLean S, Chandler D, Nurmatov U, Liu JL, Pagliari C, Car J (2010). Cochrane Database Syst Revs.

[21] Jaana M, Paré G (2007). Home telemonitoring of patients with diabetes: a systematic assessment of observed effects. J Eval Clin Pract.

[22] Craig J, Patterson V (2005). Introduction to the practice of telemedicine. J Telemed Telecare.

[23] Burns PB, Rohrich RJ, Chung KC (2011). The levels of evidence and their role in evidence-based medicine. Plast Reconstr Surg.

[24] Sackett DL, Rosenberg WM, Gray JA, Haynes RB, Richardson WS (2007). Evidence-based medicine: what it is and what it isn't. Clin Orthop Relat Res.

[25] May C, Harrison R, Finch T, MacFarlane A, Mair F, Wallace P, Telemedicine Adoption Study Group (2003). Understanding the normalization of telemedicine services through qualitative evaluation. J Am Med Inform Assoc.

[26] Tong A, Sainsbury P, Craig J (2007). Consolidated criteria for reporting qualitative research (COREQ): a 32-item checklist for interviews and focus groups. Int J Qual Health Care.

[27] Anna V, van der Ploeg HP, Cheung NW, Huxley RR, Bauman AE (2008). Sociodemographic correlates of the increasing trend in prevalence of gestational diabetes mellitus in a large population of women between 1995 and 2005. Diabetes Care.

[28] Kernan WN, Viscoli CM, Makuch RW, Brass LM, Horwitz RI (1999). Stratified randomization for clinical trials. J Clin Epidemiol.

[29] Metzger BE, Gabbe SG, Persson B, Buchanan TA, Catalano PA, Damm P, Dyer AR, Leiva AD, Hod M, Kitzmiler JL, Lowe LP, McIntyre HD, Oats JJ, Omori Y, Schmidt MI, International Association of Diabetes Pregnancy Study Groups Consensus Panel (2010). International Association of Diabetes and Pregnancy Study Group's recommendations on the diagnosis and classification of hyperglycemia in pregnancy. Diabetes Care.

[30] American Diabetes Association (2014). Standards of medical care in diabetes--2014. Diabetes Care.

[31] Online Health Portfolio.

[32] Dalfrà MG, Nicolucci A, Lapolla A (2009). The effect of telemedicine on outcome and quality of life in pregnant women with diabetes. J Telemed Telecare.

[33] Anderson RM, Fitzgerald JT, Gruppen LD, Funnell MM, Oh MS (2003). The Diabetes Empowerment Scale–Short Form (DES-SF). Diabetes Care.

[34] Attkisson C (1903). Client Satisfaction Questionnaire.

[35] Attkisson CC, Zwick R (1982). The client satisfaction questionnaire. Psychometric properties and correlations with service utilization and psychotherapy outcome. Eval Program Plann.

[36] Anderson RM, Funnell MM, Fitzgerald JT, Marrero DG (2000). The Diabetes Empowerment Scale: a measure of psychosocial self-efficacy. Diabetes Care.

[37] Zhang J, Burridge L, Baxter KA, Donald M, Foster MM, Hollingworth SA, Ware RS, Russell AW, Jackson CL (2013). A new model of integrated primary-secondary care for complex diabetes in the community: study protocol for a randomised controlled trial. Trials.

[38] Matsubara C, Green J, Astorga LT, Daya EL, Jervoso HC, Gonzaga EM, Jimba M (2013). Reliability tests and validation tests of the client satisfaction questionnaire (CSQ-8) as an index of satisfaction with childbirth-related care among Filipino women. BMC Pregnancy Childbirth.

[39] Robinson R (1993). Costs and cost-minimisation analysis. BMJ.

[40] (2012). Benefits Evaluation Survey Process–System & Use Assessment Survey.

[41] Dattakumar A, Gray K, Jury S, Biggs B, Maeder A, Noble D, Borda A, Schulz T, Gasko H (2013). A unified approach for the evaluation of telehealth implementations in Australia.

[42] (2012). Telehealth Advisory Committee Standards Framework.

[43] Maeder A, Gray K, Borda A, Poultney N, Basilakis J (2015). Achieving greater consistency in telehealth project evaluations to improve organisational learning. Stud Health Technol Inform.

[44] Hawe P, Shiell A, Riley T, Gold L (2004). Methods for exploring implementation variation and local context within a cluster randomised community intervention trial. J Epidemiol Community Health.

[45] Oakley A, Strange V, Bonell C, Allen E, Stephenson J (2006). Process evaluation in randomised controlled trials of complex interventions. BMJ.

[46] Kuijpers W, Groen WG, Aaronson NK, van Harten WH (2013). A systematic review of web-based interventions for patient empowerment and physical activity in chronic diseases: relevance for cancer survivors. J Med Internet Res.

