# Supplementary figures and images for: Telemedicine for Gestational Diabetes Mellitus (TeleGDM): A Mixed-Method Study Protocol of Effects of a Web-Based GDM Support System on Health Service Utilization, Maternal and Fetal Outcomes, Costs, and User Experience
Source: JMIR Res Protoc. 2016 Aug 9;5(3):e163. doi: 10.2196/resprot.6044 (PMC4995354; doi:10.2196/resprot.6044)

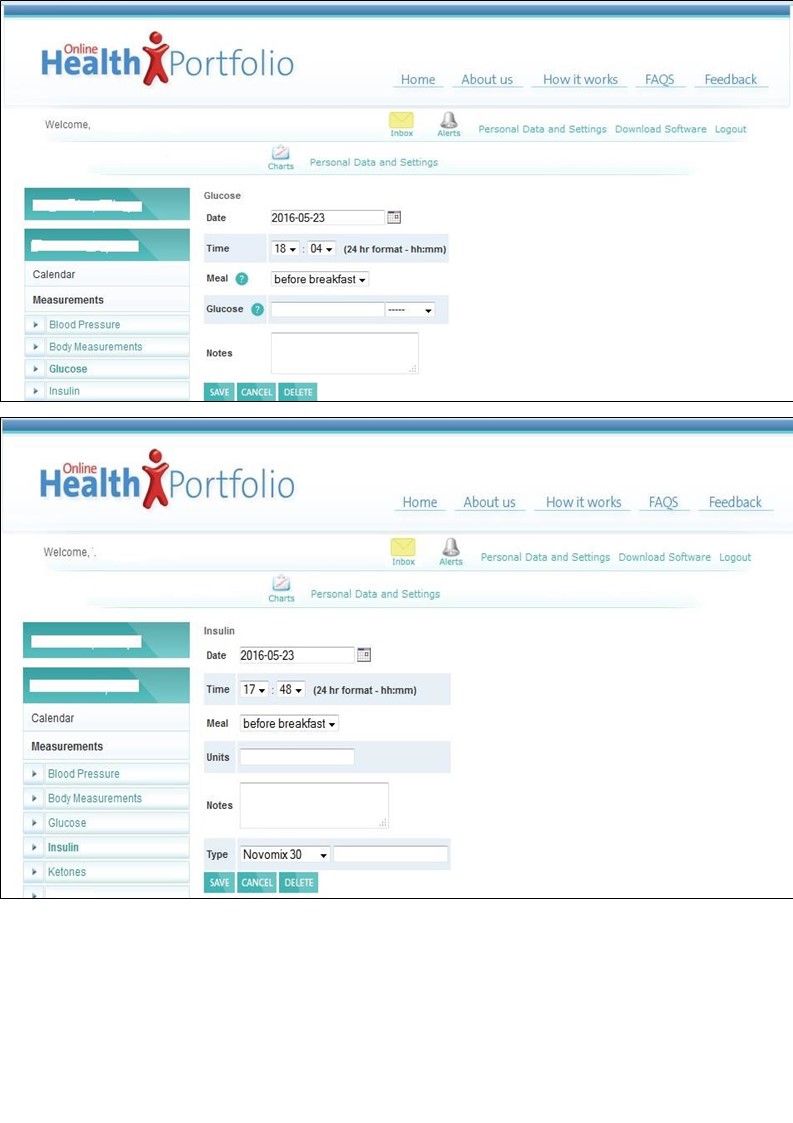

Supplement: Multimedia Appendix 1 [file resprot_v5i3e163_app1.jpg]

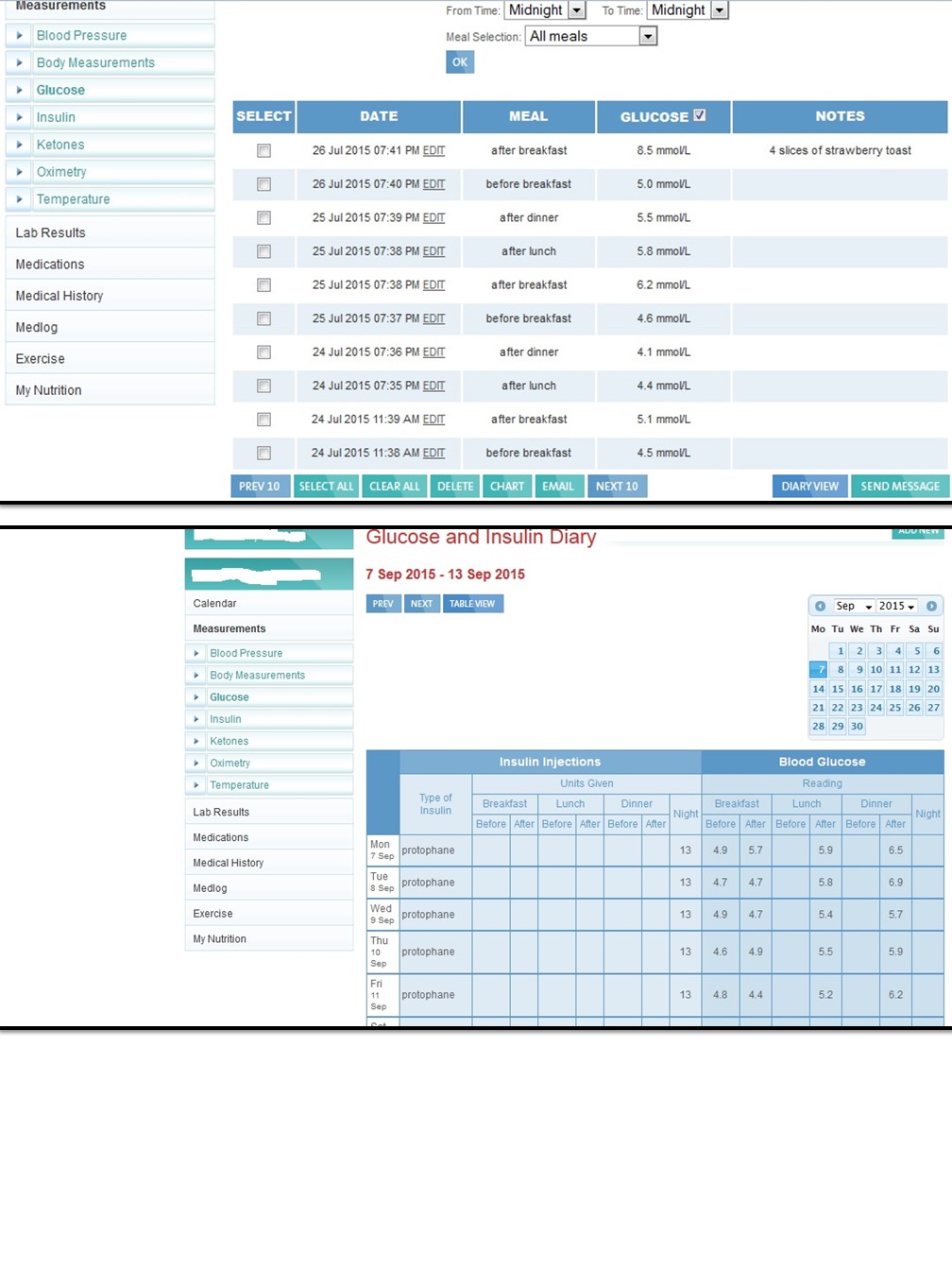

Supplement: Multimedia Appendix 2 [file resprot_v5i3e163_app2.jpg]

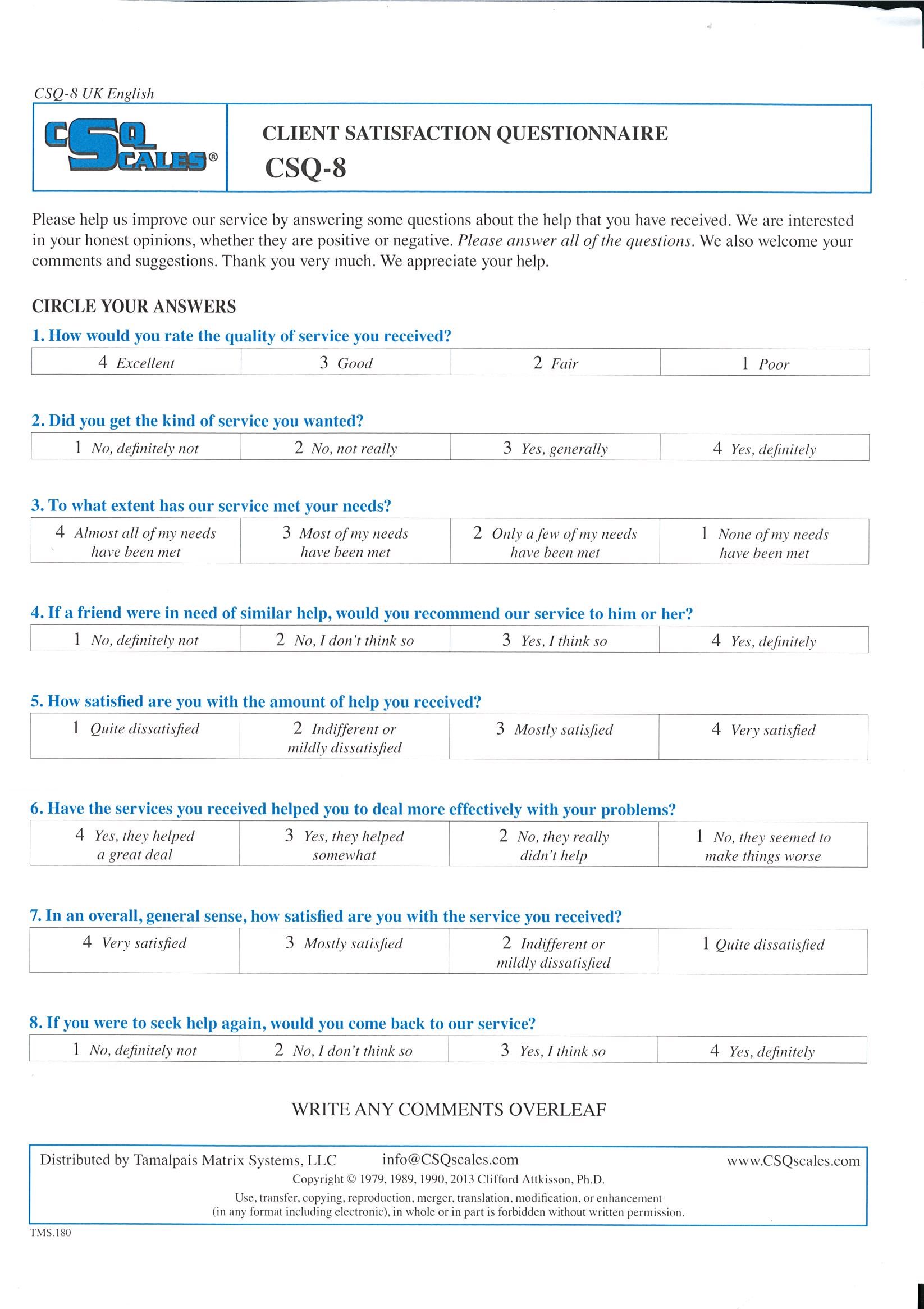

Supplement: Multimedia Appendix 4 [file resprot_v5i3e163_app4.jpg]
