# Supplementary material for: Telemedicine for Gestational Diabetes Mellitus (TeleGDM): A Mixed-Method Study Protocol of Effects of a Web-Based GDM Support System on Health Service Utilization, Maternal and Fetal Outcomes, Costs, and User Experience
Source: JMIR Res Protoc. 2016 Aug 9;5(3):e163. doi: 10.2196/resprot.6044 (PMC4995354; doi:10.2196/resprot.6044)
Supplement: Multimedia Appendix 3 [file resprot_v5i3e163_app3.pdf]

Diabetes Empowerment Scale-Short Form (DES-SF)

In general, I believe that I:

	1	2	3	4	5
1. …know what part(s) of taking care of my diabetes that I am **dissatisfied** with.	☐ Strongly Disagree	☐ Somewhat Disagree	☐ Neutral	☐ Somewhat Agree	☐ Strongly Agree
2. …am able to turn my diabetes goals into a workable plan.	☐ Strongly Disagree	☐ Somewhat Disagree	☐ Neutral	☐ Somewhat Agree	☐ Strongly Agree
3. …can try out different ways of overcoming barriers to my diabetes goals.	☐ Strongly Disagree	☐ Somewhat Disagree	☐ Neutral	☐ Somewhat Agree	☐ Strongly Agree
4. …can find ways to feel better about **having** diabetes.	☐ Strongly Disagree	☐ Somewhat Disagree	☐ Neutral	☐ Somewhat Agree	☐ Strongly Agree
5. …know the **positive** ways I cope with diabetes-related stress.	☐ Strongly Disagree	☐ Somewhat Disagree	☐ Neutral	☐ Somewhat Agree	☐ Strongly Agree
6. …can ask for support for having and caring for my diabetes when I need it.	☐ Strongly Disagree	☐ Somewhat Disagree	☐ Neutral	☐ Somewhat Agree	☐ Strongly Agree
7. …know what helps me stay motivated to care for my diabetes.	☐ Strongly Disagree	☐ Somewhat Disagree	☐ Neutral	☐ Somewhat Agree	☐ Strongly Agree
8. …know enough about myself as a person to make diabetes care choices that are right for me.	☐ Strongly Disagree	☐ Somewhat Disagree	☐ Neutral	☐ Somewhat Agree	☐ Strongly Agree
