# Supplementary material for: Telemedicine for Gestational Diabetes Mellitus (TeleGDM): A Mixed-Method Study Protocol of Effects of a Web-Based GDM Support System on Health Service Utilization, Maternal and Fetal Outcomes, Costs, and User Experience
Source: JMIR Res Protoc. 2016 Aug 9;5(3):e163. doi: 10.2196/resprot.6044 (PMC4995354; doi:10.2196/resprot.6044)
Supplement: Multimedia Appendix 5 [file resprot_v5i3e163_app5.pdf]

## Canada Health Infoway System and Use Assessment Survey

**SECTION 1. OVERALL USER SATISFACTION**

1. **In general, how satisfied are you <u>overall with the Online Health Portfolio (OH) system</u> you are currently working with or previously worked with? By "system" we mean, the ease and functionality of the OHP system itself, the quality of the information given and the quality of the services provided for the system.**

| Highly satisfied | Moderately satisfied | Neither satisfied nor dissatisfied | Moderately dissatisfied | Not at all satisfied |
|---|---|---|---|---|
| | | | | |

2. **Please indicate your level of agreement or disagreement with each of the following statements below.**

| | Strongl y | Moderatel y Agree | Moderatel y | Strongl y | Not Sur | Not Applicable |
|---|---|---|---|---|---|---|
| a.) The system improves my productivity | | | | | | |
| b.) The system improves the quality of care I can provide | | | | | | |
| c.) The system makes my job easier | | | | | | |
| d.) The system enhances our ability to coordinate the | | | | | | |
| e.) The system improves our sharing of patient information amongst providers | | | | | | |
| f.) The system enhances the efficiency of ordering lab tests, X-rays, prescriptions, | | | | | | |
| g.) The alerts, reminders and order set features (i.e. support tools) improve the quality | | | | | | |

3. **Are there aspects of the system that you would change, and if so, which ones would they be? Please describe your comments.**

4. **Do you have any experiences with the system where it has supported the provision of care? Please describe your comments.**

**SECTION 2. SYSTEM QUALITY**

5. **Based on your experiences to date with the system, how acceptable is the quality of the system itself (as described by the specific characteristics listed below)? Would you say it is;**

| Highly acceptable | Moderately acceptable | Neither acceptable nor unacceptable | Moderately unacceptable | Not at all acceptable |
|---|---|---|---|---|
| | | | | |

6. **Please indicate your level of agreement or disagreement with each of the following statements below.**

| | Strongly Agree | Moderately Agree | Moderately Disagree | Strongly Disagree | Not Sure |
|---|---|---|---|---|---|
| a.) The system is easy to use | | | | | |
| b.) The response time is acceptable | | | | | |
| c.) The system is integrated with my workflow | | | | | |
| d.) The system security is acceptable | | | | | |
| e.) The system features enable me to perform my work well | | | | | |
| f.) The system is reliable in its performance | | | | | |
| g.) Overall, the quality of the system is excellent | | | | | |

**SECTION 3: INFORMATION QUALITY**

7. **In general, when thinking about the quality of the information provided by the system, do**

**you find the quality of the information to be;**

| Highly acceptable | Moderately acceptable | Neither acceptable nor unacceptable | Moderately unacceptable | Not at all acceptable |
|---|---|---|---|---|
| | | | | |

8. **Please indicate your level of agreement or disagreement with each of the following statements below.**

| | Strongly Agree | Moderately Agree | Moderately Disagree | Strongly Disagree | Not Sure |
|---|---|---|---|---|---|
| **a)** The information is complete | | | | | |
| **b)** The information is quickly provided | | | | | |
| **c)** The information provided is accurate | | | | | |
| **d)** The information provided is relevant | | | | | |
| **e)** The information is available when I need it | | | | | |
| **f)** The format and layout of the information is acceptable | | | | | |

**SECTION 4. SERVICE QUALITY**

9. **In general, when thinking about the <u>quality of the services</u> (i.e. technical support and training services) provided for the system, do you find the quality of these services to be;**

| Highly acceptable | Moderately acceptable | Neither acceptable nor unacceptable | Moderately unacceptable | Not at all acceptable |
|---|---|---|---|---|
| | | | | |

10. **Please indicate your level of agreement or disagreement with each of the following statements below.**

: :

| | Strongly Agree | Moderately Agree | Moderately Disagree | Strongly Disagree | Not Sure |
|---|---|---|---|---|---|
| a.) The implementation process at this Hospital or Centre was acceptable | | | | | |
| b.) The current level of training is acceptable | | | | | |
| c.) The level of on-going support provided is acceptable | | | | | |

**SECTION 6. SYSTEM USAGE**

11. **In a typical day, how many times do you use the system?**

    ________________________Number of times, a day

    Always……………

    Rarely …………..

12. **In a typical week, please indicate the number of days in which you use the system.**

    ________________Number of days, a week

13. **Please estimate the percentage of your patients that you use the system for?**

    _____________% patients **(FILL IN)**

    Don't know ………….

14. **How likely are you to <u>recommend</u> the system to other healthcare providers at other Hospitals or Centres?**

| Definitely | Probably | May or may not | Probably Not | Definitely not |
|---|---|---|---|---|
| | | | | |

15. **Given a choice, would you like to increase or decrease your future use of the system that you are currently working with? Would that be a significant or moderate increase /**

**decrease, or would you like your future use to stay the same?**

| Significant Increase | Moderately Increase | Moderately Decrease | Significant Decrease | Remain the Same |
|---|---|---|---|---|
| | | | | |

**16. How would you describe your "use" of the system? (Check all that apply)**

| | |
|---|---|
| I use the system for clinical decision making ............................................... ..... | I use the system to both access patient information and in clinical decision making ...................................................... |
| I use the system to access patient information and support the clinical decision maker | |

**SECTION 7. OTHER COMMENTS**

**17. Do you have any other comments you would like to make regarding the system?**
