# Supplementary material for: Telemedicine for Gestational Diabetes Mellitus (TeleGDM): A Mixed-Method Study Protocol of Effects of a Web-Based GDM Support System on Health Service Utilization, Maternal and Fetal Outcomes, Costs, and User Experience
Source: JMIR Res Protoc. 2016 Aug 9;5(3):e163. doi: 10.2196/resprot.6044 (PMC4995354; doi:10.2196/resprot.6044)
Supplement: Multimedia Appendix 6 [file resprot_v5i3e163_app6.pdf]

## Interview Schedule

### A. Patient Interview Questions

As you might recall you were a participant in the TeleGDM study, and used Online Health Portfolio to help with managing diabetes during pregnancy. The purpose of this interview is to get **as much of your story as possible on how you found using OHP to manage the diabetes and how you found the OHP itself. So there are no right or wrong answers; it's about your views and experiences** noting we have no personal/vested interests in OHP.

1. So to start off tell me about managing GDM itself, how do you think you went with
    a. Managing the diabetes
    b. Insulin
    c. Getting help from the clinician; who was involved?
2. (the good, the bad or the not so good what worked or didn't work for you)
3. Let's focus on the technology/OHP itself. How did that go (the good, the bad or the not so good what worked or didn't work for you)
    a. How it helped or didn't help you manage GDM
    b. Getting support from the clinicians
    c. Attending appointments
    d. Entering data (frequency, confidence, convenience)
    e. What did you use to enter you data, **(Smartphone, tablet, Windows computer, Mac)?**
4. Tell me about any **problems or issues** you came across using OHP (what were these problems and how did you deal with (**resolve)** them?)
5. What **suggestions** do you have which might help us improve providing GDM management and support via technology like OHP?
6. If in the **future**, if you were given the opportunity to use telemedicine to support you manage GDM or use the usual way, which would you **choose** and why?
    a. While we provided OHP free for you to use during that time, what impact would **cost** have on your choice/decision whether to use technology like OHP?
    b. What other factors would influence your choice?
7. I have a few more general background questions to finish with…If it's alright,
    - Have you had experience with using technology for health, work or on a personal level? (e.g. This may include health consultations, sharing health information, making appointments online, self-monitoring devices e.g. fitbit and the like etc.)
    - How far do you live from the clinic?
    - What is the highest level of education you have achieved?

### B. Clinician (CDE-RN) Interview Questions

As a clinician who had the opportunity to use telemedicine/telehealth in the TeleGDM trial your views on this approach are very valuable. I would like to hear of your experiences, i.e. the good, the bad and the not so good about using telehealth to support women with GDM. These are experiences

about providing clinical care, impact on your workflow, usability of the technology (OHP) and its features, how your patients used the telehealth system and how you interacted with them.

The following questions are only a guide but are not intended to limit your views. Feel free to provide as much information and details as possible.

1. During the TeleGDM trial you used a telemedicine/telehealth using OHP to support women with GDM. Can you tell me:
    a. Your overall experience with using telemedicine for the management of GDM?
    b. How confident did you feel accessing data online?
    c. Was using OHP convenient or inconvenient and whether it was or not, how so?
    d. What problems and frustrations did you encounter and how did you deal with them?
2. What are your views of:
    a. Using telemedicine (OHP) compared usual care?
    b. How different or similar was this to usual care?
3. How did using telehealth impact your workflow, patient scheduling and clinical decision making?
4. How often did you:
    a. Access the system?
    b. Interact with patients over the system, i.e. messaging?
    c. Your views on safety and effectiveness of providing care and support via telemedicine?
5. Is the telemedicine an approach for everyone or what groups of patients could this be suitable for?
6. What you think would make clinicians embrace and take up telemedicine in GDM care?
7. If in the future, you had the option to provide care and support via telemedicine or usual care,
    a. What would choose and what factors would you consider in making the decision/choice?
8. Going into the future what are your suggestions on how we can improve providing GDM management support via telemedicine and what elements/features or improvements of the online system do you think might make it better/easier and useful?
9. Please tell me about any other thoughts you haven’t expressed above.
10. Finally, are you okay for me to contact you for some follow-up question?
